# Reliability of the classification of cartilage and labral injuries during hip arthroscopy

**DOI:** 10.1093/jhps/hnaa064

**Published:** 2021-03-06

**Authors:** Stephanie W Mayer, Tobias R Fauser, Robert G Marx, Anil S Ranawat, Bryan T Kelly, Stephen Lyman, Danyal H Nawabi

**Affiliations:** 1 Department of Orthopedic Surgery, University of Colorado, 175 Inverness Dr W, Ste. 400 Englewood, CO 80112, USA; 2 College of Medicine – Tucson, University of Arizona, 1501 N Campbell Ave, Tucson, AZ 85724, USA; 3 Department of Orthopedic Surgery, Hospital for Special Surgery, 535 East 70th Street, New York, NY 10021, USA; 4 Research Institute, Hospital for Special Surgery, 535 East 70th Street, New York, NY 10021, USA

## Abstract

To determine interobserver and intraobserver reliabilities of the combination of classification systems, including the Beck and acetabular labral articular disruption (ALAD) systems for transition zone cartilage, the Outerbridge system for acetabular and femoral head cartilage, and the Beck system for labral tears. Additionally, we sought to determine interobserver and intraobserver agreements in the location of injury to labrum and cartilage. Three fellowship trained surgeons reviewed 30 standardized videos of the central compartment with one surgeon re-evaluating the videos. Labral pathology, transition zone cartilage and acetabular cartilage were classified using the Beck, Beck and ALAD systems, and Outerbridge system, respectively. The location of labral tears and transition zone cartilage injury was assessed using a clock face system, and acetabular cartilage injury using a five-zone system. Intra- and interobserver reliabilities are reported as Gwet’s agreement coefficients. Interobserver and intraobserver agreement on the location of acetabular cartilage lesions was highest in superior and anterior zones (0.814–0.914). Outerbridge interobserver and intraobserver agreement was >0.90 in most zones of the acetabular cartilage. Interobserver and intraobserver agreement on location of transition zone lesions was 0.844–0.944. The Beck and ALAD classifications showed similar interobserver and intraobserver agreement for transition zone cartilage injury. The Beck classification of labral tears was 0.745 and 0.562 for interobserver and intraobserver agreements, respectively. The Outerbridge classification had almost perfect interobserver and intraobserver agreement in classifying chondral injury of the true acetabular cartilage and femoral head. The Beck and ALAD classifications both showed moderate to substantial interobserver and intraobserver reliabilities for transition zone cartilage injury. The Beck system for classification of labral tears showed substantial agreement among observers and moderate intraobserver agreement. Interobserver agreement on location of labral tears was highest in the region where most tears occur and became lower at the anterior and posterior extents of this region. The available classification systems can be used for documentation regarding intra-articular pathology. However, continued development of a concise and highly reproducible classification system would improve communication.

## INTRODUCTION

The concept of femoroacetabular impingement (FAI) as described by Ganz et al. led to an improved understanding of mechanical forces that can lead to labral and chondral injury and early arthritis [1, 2]. Bony morphology of the femur and acetabulum has been shown to be associated with the intra-articular injury pattern of the labrum and cartilage seen on imaging and during surgery [3]. With this knowledge, open and arthroscopic procedures for the treatment of FAI have been developed [4–6]. Recently, there has been a focus on hip arthroscopy to treat FAI, and with this focus, an increase in the volume of scientific research on this procedure [7, 8]. Generally, good to excellent outcomes are reported following hip arthroscopy for FAI [9]; however, optimal patient characteristics for this procedure are not completely defined. Severity of injury to intra-articular structures such as the labrum and articular cartilage is associated with inferior outcomes in some studies [10–13]. A method to consistently communicate arthroscopic findings among clinicians and researchers is important.

There are several described classification systems for cartilage lesions. The Outerbridge system [14] has been used extensively [10, 15–17]. Specific to the hip, there are three described systems which focus on the transition zone cartilage at the periphery of the acetabulum which is commonly injured in FAI. The Beck classification was developed to classify transition zone cartilage injury seen during surgical hip dislocation and can also be used during arthroscopy [3, 18]. The Haddad and acetabular labral articular disruption (ALAD) classifications were developed for transition zone articular cartilage injury as seen during hip arthroscopy [19, 20]. Although several labral injury classifications are described [21–23], currently the labral injury pattern is most commonly discussed using the Beck classification [3].

Three articles have tested interobserver reliability of the Beck, Haddad and Outerbridge classification systems for cartilage and the Beck system for labral tears with varying levels of agreement [19, 24, 25]. Reliability of the ALAD classification has not been previously reported. Therefore, the purpose of this study was to determine interobserver and intraobserver reliabilities of the combination of classification systems, including the Beck and ALAD systems for transition zone cartilage, the Outerbridge system for acetabular and femoral head cartilage, and the Beck system for labral tears. Additionally, we sought to determine interobserver and intraobserver agreements in the location of injury to labrum and cartilage. We hypothesized that there would be good correlation between surgeons in classifying the grade of cartilage and labral injury, but that there would be fair or poor agreement in the location of the tear.

## MATERIALS AND METHODS

**Fig. 1.**
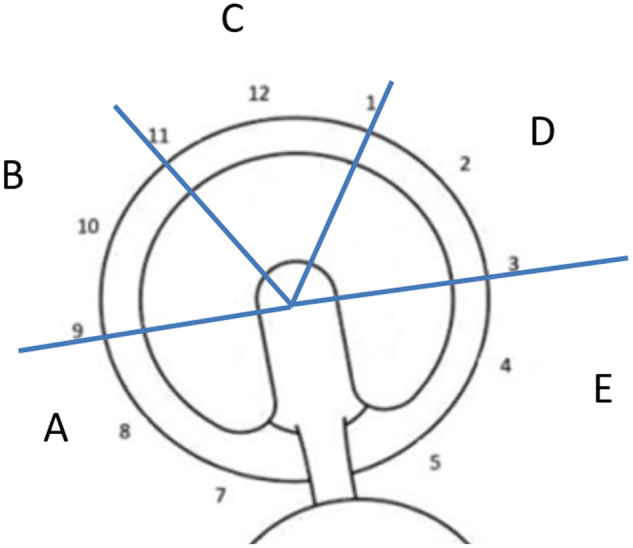
Five-zone system for location of true and transition zone acetabular cartilage injury.

Thirty standardized videos of the intra-operative arthroscopic assessment of the central compartment of the hip during primary hip arthroscopy were reviewed by three surgeon observers. This sample size was chosen based on a power analysis optimized for the number of observers [26]. All arthroscopies were performed by a single surgeon, in supine, using anterolateral and modified anterior portals [27]. Each video was performed with the arthroscope in the anterolateral portal following an interportal capsulotomy for visualization. Videos were screened prior to viewing by authors other than the observers to ensure that the videos followed the same progression of inspection of the joint, were of high quality, and provided the necessary views. Labrum, transition zone cartilage, true acetabular cartilage and femoral head cartilage were visualized with two to three passes across the entirety of the visible structure. The three faculty-ranked observers were at different stages of their orthopedic surgery careers. All observers were fellowship trained hip arthroscopists. One had been in practice over 10 years, one between 5 and 10 years, and one between 1 and 5 years. Observers independently watched the videos, with one immediate repeat viewing if requested. For evaluation of the labrum, the Beck classification was used (Table I) [3]. For transition zone cartilage injury (defined as the 5 mm of acetabular cartilage just deep to the chondrolabral junction) [28], the ALAD [20] and Beck [3] systems were used (Tables II and III). For true acetabular cartilage and femoral head, the Outerbridge system was used (Table IV) [14]. Location of labral tears was reported using a clock face system, with 3 o′clock being anterior. Location of true acetabular cartilage injury and transition zone cartilage injury was reported using a five-zone system, A–E, where E is most anterior (Fig. 1). Observers were provided the same description of the labral and chondral classification systems and location zones prior to evaluation. A standardized form was completed by observers for each video including each of these data points (Supplementary Appendix S1).

**Table I.** Beck classification of labral tears

Stage		Labral status
0	Normal	Macroscopically normal
1	Degeneration	Thinning or localized hypertrophy, fraying, discoloration
2	Full thickness tears	Complete separation from rim
3	Detachment	Separation between acetabular and labral cartilage, preserved attachment to bone
4	Ossification	Osseous metaplasia, localized or circumferential

**Table II.** ALAD classification of transition zone cartilage injury

Stage	Articular cartilage status
0	Macroscopically normal
1	Softening
2	Fissures
3	Flap
4	Exposed Bone

**Table III.** Beck classification of transition zone cartilage injury

Stage		Articular cartilage status
0	Normal	Macroscopically sound
1	Malacia	Roughening of surface, fibrillation
2	Debonding	Loss of fixation to subchondral bone, macroscopically sound cartilage, carpet phenomenon, wave sign
3	Cleavage	Loss of fixation to subchondral bone, frayed edges, thinning of cartilage, flap
4	Defect	Full thickness defect

**Table IV.** Outerbridge classification of cartilage defects

Grade	
0	Normal cartilage
I	Softening
II	Superficial, partial thickness fissures
III	Full thickness fissures
IV	Exposed subchondral bone

Inter- and intra-rater reliabilities between all three raters are reported as Gwet’s agreement coefficient (AC) [29] [first-order AC (AC_1_) for binary variables and second order AC (AC_2_) for ordinal variables] along with Fleiss’s kappa [30] (simple kappa for binary variables and weighted kappa for ordinal variables) and proportion agreement (raw proportion for binary variables and weighted proportion for ordinal variables). Ordinal weights were used to calculate all weighted agreement measures. Agreement measures are reported as point estimates with associated standard error. Strength of agreement was interpreted as follows: < 0.00 = poor, 0.00–0.20 = slight, 0.21–0.40 = fair, 0.41–0.60 = moderate, 0.61–0.80 = substantial, 0.81–0.99 = almost perfect and 1.0 = perfect [31]. Statistical analyses were performed with SAS Version 9.3 (SAS Institute, Cary, NC).

## RESULTS

**Fig. 2.**
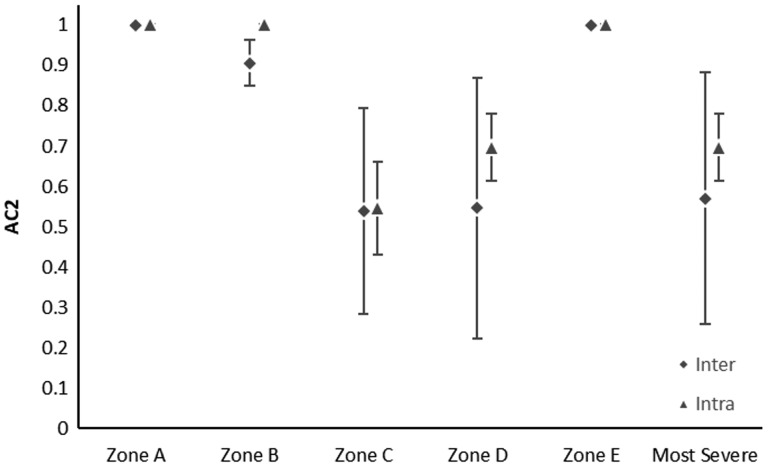
Scatter plot showing the interobserver (diamonds) and intraobserver (triangles) agreement plus or minus one standard error for ALAD classification of transition zone cartilage lesion.

**Fig. 3.**
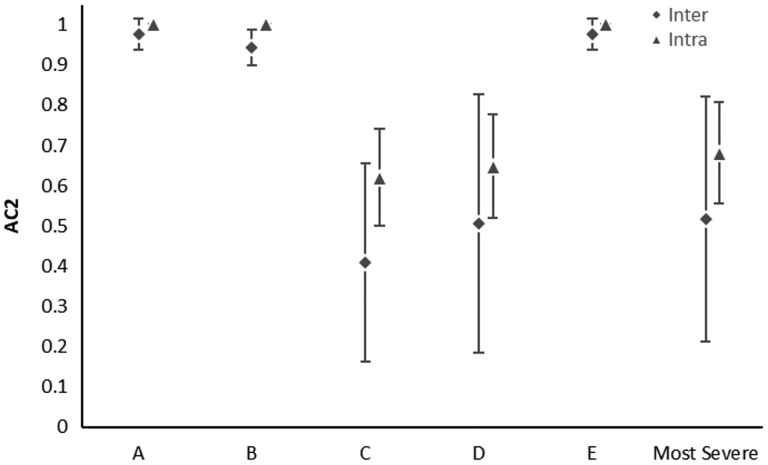
Scatter plot showing the interobserver (diamonds) and intraobserver (triangles) agreement plus or minus one standard error for Beck classification of cartilage lesion.

**Fig. 4.**
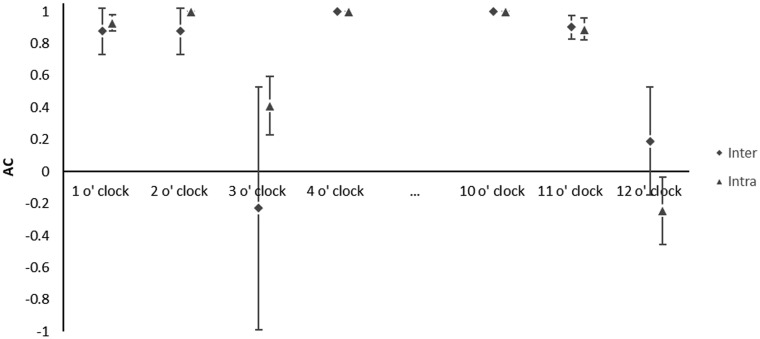
Scatter plot showing the interobserver (diamonds) and intraobserver (triangles) agreements plus or minus one standard error for location of labral tear noted along clock face.

Thirty hip surgeries on 28 patients (25% female, mean age 30.0 years ±11.8 years, range: 15.2–55.8 years) were reviewed. Results of interobserver and intraobserver agreements of each classification are noted in Tables V–VII. Agreement was almost perfect or perfect (> 0.926) in determining the location of acetabular cartilage injury in zones A–E (Tables V and VI). There was almost perfect or perfect agreement using the Outerbridge classification for the type of acetabular cartilage defect in each zone (> 0.926) (Tables V and VI). The location of transition zone chondral injury showed almost perfect or perfect agreement in all zones (> 0.844) (Tables V and VI). Moderate to substantial agreement was found in classifying the type of transition zone cartilage injury in Zones C and D using the ALAD system (0.538–0.695) and almost perfect to perfect in all other zones with similar results for the Beck system (Tables V and VI, Figs. 2 and 3). The single most severe transition zone injury classified overall by observers showed moderate interobserver agreement using both the ALAD system (0.571) and the Beck system (0.517), while intraobserver agreement was substantial (0.695 and 0.679) for the respective systems. Absence of a labral tear was noted between 4 and 10 o′clock by all raters in all cases (Table VII). Agreement in the presence of a labral tear was highest between 1 and 2 o′clock, and at 11 o′clock, with substantial to almost perfect correlation (0.876–0.929) (Table VII, Fig. 4). Poor interobserver agreement at 3 o′clock was noted (−0.229), while intraobserver agreement was moderate (0.412) (Table VII, Fig. 4). Substantial interobserver agreement was found in classifying the type of labral tear with the Beck system (0.745) with only moderate intraobserver agreement (0.562) (Table VII).

**Table V.** Interobserver reliability of Outerbridge classification for acetabular articular cartilage and femoral head chondral defects, and ALAD and Beck transition zone chondral injury rated by zone and the most severe rating noted

	Presence of cartilage defect				Rating of cartilage defect		
Variable	AC1		Simple kappa	Proportion agreement	AC2	Weighted kappa	Weighted proportion agreement
Outerbridge classification of acetabular articular chondral defect							
Zone A	0.952 (0.042)		0.310 (0.478)	0.956 (0.038)	0.957 (0.047)	0.190 (0.314)	0.959 (0.043)
Zone B	0.977 (0.033)		−0.011 (0.011)	0.978 (0.031)	0.977 (0.033)	−0.011 (0.011)	0.978 (0.031)
Zone C	N/A		N/A	1 (0)	N/A	N/A	1 (0)
Zone D	0.977 (0.033)		−0.011 (0.011)	0.978 (0.031)	0.977 (0.033)	−0.011 (0.011)	0.978 (0.031)
Zone E	N/A		N/A	1 (0)	N/A	N/A	1 (0)
Most severe	—		—	—	0.957 (0.047)	0.19 (0.314)	0.959 (0.043)
Outerbridge classification of femoral chondral defect							
Zone A (anterior)	0.954 (0.042)		−0.023 (0.02)	0.956 (0.038)	0.954 (0.042)	−0.023 (0.02)	0.956 (0.038)
Zone B (superior)	N/A		N/A	1 (0)	N/A	N/A	1 (0)
Zone C (anterior)	N/A		N/A	1 (0)	N/A	N/A	1 (0)
Zone D (anterior)	N/A		N/A	1 (0)	N/A	N/A	1 (0)
Zone E (anterior)	N/A		N/A	1 (0)	N/A	N/A	1 (0)
Worst	—		—	—	0.954 (0.042)	−0.023 (0.02)	0.956 (0.038)
ALAD classification of transition zone cartilage lesions							
Zone A		N/A	N/A	1 (0)	N/A	N/A	1 (0)
Zone B		0.844 (0.111)	0.071 (0.208)	0.867 (0.083)	0.906 (0.056)	0.094 (0.225)	0.919 (0.041)
Zone C		0.929 (0.061)	−0.034 (0.029)	0.933 (0.053)	0.538 (0.255)	0.175 (0.262)	0.826 (0.084)
Zone D		0.954 (0.058)	−0.023 (0.016)	0.956 (0.054)	0.547 (0.323)	0.119 (0.391)	0.826 (0.111)
Zone E		N/A	N/A	1 (0)	N/A	N/A	1 (0)
Most severe		—	—	—	0.571 (0.313)	0.159 (0.383)	0.833 (0.108)
Beck classification of transition zone cartilage lesions							
Transition zone A		0.977 (0.039)	−0.011 (0.011)	0.978 (0.038)	0.977 (0.039)	−0.011 (0.011)	0.978 (0.038)
Transition zone B		0.814 (0.205)	0.040 (0.226)	0.844 (0.155)	0.943 (0.044)	0.068 (0.226)	0.952 (0.031)
Transition zone C		0.903 (0.056)	−0.047 (0.026)	0.911 (0.048)	0.41 (0.247)	0.014 (0.359)	0.785 (0.082)
Transition zone D		0.977 (0.033)	−0.011 (0.011)	0.978 (0.031)	0.506 (0.322)	0.031 (0.551)	0.811 (0.119)
Transition zone E		0.977 (0.039)	−0.011 (0.011)	0.978 (0.038)	0.977 (0.039)	−0.011 (0.011)	0.978 (0.038)
Most severe		—	—	—	0.517 (0.304)	0.050 (0.500)	0.815 (0.110)

Gwet’s AC and Fleiss’ kappa analysis. Agreement measures are shown as point estimate (standard error). N/A is indicated when all observers rated the variable as ‘No’ or ‘Normal’.

**Table VI.** Intraobserver reliability of Outerbridge classification for acetabular articular cartilage and femoral head chondral defects, and ALAD and Beck transition zone chondral injury rated by zone and the most severe rating noted

	Presence of cartilage defect			Rating of cartilage defect		
Variable	AC1	Simple kappa	Proportion agreement	AC2	Weighted kappa	Weighted proportion agreement
Outerbridge classification of acetabular articular chondral defect						
Zone A	0.926 (0.063)	−0.07 (0.042)	0.931 (0.058)	0.926 (0.063)	−0.07 (0.042)	0.931 (0.058)
Zone B	N/A	N/A	1 (0.034)	N/A	N/A	1 (0.034)
Zone C	N/A	N/A	1 (0.034)	N/A	N/A	1 (0.034)
Zone D	N/A	N/A	1 (0.034)	N/A	N/A	1 (0.034)
Zone E	N/A	N/A	1 (0.034)	N/A	N/A	1 (0.034)
Most severe	—	—	—	0.926 (0.063)	−0.070 (0.042)	0.931 (0.058)
Outerbridge classification of femoral chondral defect						
Zone A (anterior)	N/A	N/A	1 (0)	N/A	N/A	1 (0)
Zone B (superior)	N/A	N/A	1 (0)	N/A	N/A	1 (0)
Zone C (anterior)	N/A	N/A	1 (0)	N/A	N/A	1 (0)
Zone D (anterior)	N/A	N/A	1 (0)	N/A	N/A	1 (0)
Zone E (anterior)	N/A	N/A	1 (0)	N/A	N/A	1 (0)
Worst	—	—	—	N/A	N/A	1 (0)
ALAD classification of transition zone cartilage lesions						
Zone A	N/A	N/A	1 (0.034)	N/A	N/A	1 (0.034)
Zone B	N/A	N/A	1 (0.034)	N/A	N/A	1 (0.034)
Zone C	N/A	N/A	1 (0.034)	0.545 (0.115)	0.313 (0.149)	0.805 (0.051)
Zone D	N/A	N/A	1 (0.034)	0.695 (0.083)	0.470 (0.130)	0.862 (0.043)
Zone E	N/A	N/A	1 (0.034)	N/A	N/A	1 (0.034)
Most severe	—	—	—	0.695 (0.083)	0.470 (0.130)	0.862 (0.043)
Beck classification of transition zone cartilage lesions						
Transition zone A	N/A	N/A	1 (0)	N/A	N/A	1 (0)
Transition zone B	N/A	N/A	1 (0)	N/A	N/A	1 (0)
Transition zone C	0.890 (0.068)	−0.053 (0.031)	0.900 (0.056)	0.619 (0.120)	0.373 (0.160)	0.850 (0.043)
Transition zone D	N/A	N/A	1 (0)	0.647 (0.128)	0.532 (0.149)	0.844 (0.050)
Transition zone E	N/A	N/A	1 (0)	N/A	N/A	1 (0)
Most severe	—	—	—	0.679 (0.126)	0.566 (0.146)	0.856 (0.050)

Gwet’s AC and Fleiss’ kappa analysis. Agreement measures are shown as point estimate (standard error).

**Table VII.** Interobserver and intraobserver reliability of Beck classification for labral tears

	Interobserver agreement			Intraobserver agreement		
Variable	AC (SE)	Kappa (SE)	Proportion agreement (SE)	AC (SE)	Kappa (SE)	Proportion agreement (SE)
Labral tear Beck classification^a^	0.745 (0.078)	0.208 (0.301)	0.900 (0.030)	0.562 (0.103)	0.234 (0.160)	0.811 (0.040)
Clock face labral tear (yes/no)						
1 o′clock	0.876 (0.143)	−0.059 (0.026)	0.889 (0.120)	0.929 (0.053)	−0.034 (0.025)	0.933 (0.046)
2 o′clock	0.876 (0.143)	−0.059 (0.026)	0.889 (0.120)	N/A	N/A	1 (0)
3 o′clock	−0.229 (0.758)	−0.260 (0.437)	0.378 (0.322)	0.412 (0.181)	−0.250 (0.071)	0.600 (0.091)
4–10 o′clock	N/A	N/A	1 (0)	N/A	N/A	1 (0)
11 o′clock	0.901 (0.074)	0.153 (0.374)	0.911 (0.061)	0.890 (0.068)	−0.053 (0.031)	0.900 (0.056)
12 o′clock	0.199 (0.336)	0.016 (0.252)	0.556 (0.147)	−0.245 (0.211)	−0.435 (0.141)	0.333 (0.088)

Agreement measures are shown as point estimate (standard error). N/A is indicated when all observers rated the variable as ‘No’ or ‘Normal’.

a Weighted agreement measure.

## DISCUSSION

The chondral pathology noted in hip conditions such as FAI have been classified in several systems. The Outerbridge system was originally described for cartilage injury in the knee [14] but has been adapted to the hip (Table IV) [10, 15–17]. Later, Beck et al. described the association between bony morphology seen in FAI and the pattern of chondral and labral injury seen during surgery [3]. With FAI, particularly cam type FAI, a specific pattern of injury to the outer margin of cartilage and chondrolabral junction, the transition zone, is noted. Chondral injury in this region is caused by the shearing forces of the cam lesion as it enters the joint [1] and follows a consistent progression. First, softening of the cartilage is seen, followed by debonding of the cartilage from the underlying acetabular bone, a cleavage at the chondrolabral junction leaving a loose flap of cartilage, and finally a complete defect or void if this cartilage flap breaks free. This chondral injury pattern of the transition zone does not follow the same progression through the stages of the Outerbridge classification, and therefore other classifications such as the Beck, Haddad and ALAD (Tables II and III) systems were developed to account for this difference [3, 19, 20]. The most widely used classification system for labral tears is the Beck system [3]. This system takes into account the type of mechanical force which may have caused the labral injury and is not on a progressive spectrum (Table I).

Three articles have tested intra- and interobserver reliability of combinations of the Beck, Haddad and Outerbridge classification systems for cartilage and the Beck system for labral tears with varying results. One article has tested the intra- and interobserver reliability of reporting the location of labral and chondral injuries [32]. This study expands on prior work by better describing the combination of classification of injury and location of pathology for a comprehensive examination of the joint.

Additionally, we chose to report reliabilities using Gwet’s AC rather than the traditional Kappa values reported in previous studies. Kappa is a widely used AC that is adjusted for the degree of agreement that would be expected solely by chance. However, unexpectedly low kappa values result when there is very high or very low trait prevalence or there is good agreement between raters on marginal counts [33]. We present Gwet’s AC as our main measures of agreement because they are more resistant to these paradoxes than kappa [29]. The previous studies which have reported the interobserver and intraobserver agreements of the classifications of cartilage and labral injury overall and found a range of agreement from fair to substantial and the study on location found poor agreement [19, 24, 25, 32]. We sought to determine interobserver and intraobserver reliabilities of each part of this comprehensive intra-operative analysis.

We found almost perfect to perfect agreement in the presence and grading of acetabular and femoral head chondral lesions using the Outerbridge classification. Recently, Amenabar et al. reported interobserver reliability of the Outerbridge, Beck and Haddad classifications for acetabular cartilage lesions among four orthopedic surgeons [25]. They found fair agreement using the Outerbridge (average *k* = 0.28) and Beck (average *k* = 0.33) classifications, and moderate agreement using the Haddad classification (average *k* = 0.47). Absolute agreement was noted in 12.5% of cases when using the Outerbridge system, 20% using the Beck system and 40% using the Haddad system. Intraobserver agreement was substantial using all three systems (*k* = 0.62–0.68). The higher agreement noted in our study may be multifactorial including the number and types of chondral lesions included in each study, bias of surgeons and statistical analysis methods.

Agreement in the presence or absence of transition zone cartilage injury was almost perfect or perfect for all zones. Agreement in the classification of the transition zone injury, however, was roughly inverse to the typical pattern of frequency in transition zone cartilage injury. Zones C and D (anterior–superior) are most commonly involved, followed by zones B and E, and finally zone A. Our results found that in the region of most common pathology, there was the lowest agreement in the classification of transition zone injury. Agreement remained moderate in these regions; however, this suggests that work should be done to create a more reliable classification, as it seems that when there is pathology present, these classification systems are not as reproducible.

The third way we analyzed agreement in transition zone injuries was using the single most severe injury classification noted overall by observers. Agreement was substantial using the ALAD and Beck systems. The study by Nepple utilized one transition zone classification per hip and therefore this portion of our analysis may be more appropriately compared to this study. Using the weighted Cohen kappa value, Nepple et al. found substantial interobserver reliability between three orthopedic surgeons using the Beck classification of transition zone cartilage injury (*k* = 0.65) [24]. Absolute agreement occurred in only 32.5% of the cartilage injury cases, however. Amenabar grouped all cartilage injury together including true and transition zone, however, they reported fair to moderate interobserver reliability as above [25]. Konan et al. studied interobserver agreement of the Haddad system. This classification takes into account the location and type of injury to the acetabular cartilage, creating a different class for each combination that encompasses the transition zone and true acetabular cartilage [19]. Using the intraclass correlation coefficient (ICC), they found almost perfect agreement overall between observers with an ICC of 0.88, although they did not discuss differences in agreement across different locations of the acetabulum which may account for differences from our results.

The class of labral tear using the Beck system had substantial interobserver agreement and moderate intraobserver agreement. The most common disagreement was between the classes of degeneration and detachment, followed by full thickness tear and detachment. This likely occurs due to the inherent limitations of the Beck system. This system attempts to classify labral injury based on the mechanical pattern that caused the injury and does not follow a spectrum of disease from benign to severe, as a labrum which is degenerative and a ‘2’ in this system is commonly more severely injured than a detached labrum which is classified as a ‘4’. In cam type FAI, pincer type FAI and hip dysplasia, the pattern of injury to the labrum is clearly different, and there are early and late forms of injury in each. A separate classification scheme for each type may be useful in the future. Nepple et al. found substantial interobserver reliability between three orthopedic surgeons using the Beck classification of labral tears (*k* = 0.62). Absolute agreement was seen in 67.5% of labral tears. Similar to our results, they found that degeneration versus detachment was the most common discrepancy and concluded that a labral tear classification with a progression of severity specific to each mechanical derangement of the hip would be more appropriate. Separate classifications which are unique to the stages of injury seen with each type of mechanical stress on labrum and cartilage (i.e. separate classifications for cam versus pincer FAI) may yield higher interobserver reliability.

The most common location of labral injury in FAI is in the anterior–superior region of the acetabulum, or 12–2 o′clock using a clock face system [3]. Previous work has found that the description of location of labral pathology had poor to fair reliability between surgeons [32]. Our results indicate that agreement of the presence of a tear is excellent in the central part of this common region, but agreement on the anterior and posterior extent of the tear was poor, suggesting that determining the anterior and posterior extent of the zone of labral injury is highly variable among surgeons. In the 4 o′clock to 10 o′clock regions where labral tears are less common, agreement was perfect, given the infrequency of lesions which are notable to surgeons when present.

Our study has several limitations. First, we are limited to hips which were clinically indicated for arthroscopy. Therefore, observers may have been biased toward classifying labral and chondral pathology according to the typical patterns seen at arthroscopy. The operating surgeon was also an observer, though the surgeon is a high volume arthroscopist and a minimum of 6 months passed between performing the surgery and being shown a blinded video of the case. The surgeon was unable to correctly identify any of the 28 patients based on viewing the video alone nor was the surgeon able to correctly identify which patients were bilateral cases. The two surgeons with less experience were trained by the senior surgeon, which may influence the way each surgeon interpreted the videos. Our results may not be generalizable to all orthopedic surgeons who have not been trained to interpret intra-articular pathology in the same systematic fashion. A final limitation is that one surgeon participated in intraobserver reliability.

Strengths of the study include the addition of the interobserver and intraobserver reliabilities of both location and classification of chondral and labral pathology to the literature rather than just presence or absence. Additionally, this is the first study to report the reliability of the ALAD classification. The observers in this study were of three varying levels of experience, and therefore our results represent the same levels of experience found among the population of surgeons treating this pathology. Our results may then be translated to the agreement we would expect among surgeons in practice.

## CONCLUSION

The Outerbridge classification had almost perfect interobserver and intraobserver agreement in classifying chondral injury of the true acetabular cartilage and femoral head. The Beck and ALAD classifications both showed moderate to substantial interobserver and intraobserver reliabilities for transition zone cartilage injury. The Beck system for classification of labral tears showed substantial agreement among observers and moderate intraobserver agreement. Interobserver agreement on location of labral tears was highest in the region where most tears occur and became lower at the anterior and posterior extents of this region.

## SUPPLEMENTARY DATA

Supplementary data are available at *Journal of Hip Preservation Surgery* online.

## Supplementary Material

hnaa064_Supplementary_Data

## References

[1] Ganz R , Parvizi J, Beck M et al Femoroacetabular impingement: a cause for osteoarthritis of the hip. Clin Orthop Relat Res 2003; 417: 112–20. 10.1097/01.blo.0000096804.78689.c2 14646708

[2] Leunig M , Beaule PE, Ganz R. The concept of femoroacetabular impingement: current status and future perspectives. Clin Orthop Relat Res 2009; 467: 616–22. 19082681 10.1007/s11999-008-0646-0 PMC2635437

[3] Beck M , Kalhor M, Leunig M et al Hip morphology influences the pattern of damage to the acetabular cartilage: femoroacetabular impingement as a cause of early osteoarthritis of the hip. J Bone Joint Surg Br 2005; 87-B: 1012–8. 10.1302/0301-620X.87B7.15203 15972923

[4] Ganz R , Gill TJ, Gautier E et al Surgical dislocation of the adult hip a technique with full access to the femoral head and acetabulum without the risk of avascular necrosis. J Bone Joint Surg Br 2001; 83-B: 1119–24. 10.1302/0301-620x.83b8.11964 11764423

[5] Byrd JW. Hip arthroscopy utilizing the supine position. Arthroscopy 1994; 10: 275–80. 8086020 10.1016/s0749-8063(05)80111-2

[6] Glick JM. Hip arthroscopy using the lateral approach. Instr Course Lect 1988; 37: 223–31. 3418122

[7] Sing DC , Feeley BT, Tay B et al Age-related trends in hip arthroscopy: a large cross-sectional analysis. Arthroscopy 2015; 31: 2307–13.e2. 26194938 10.1016/j.arthro.2015.06.008

[8] Degen RM , Bernard JA, Pan TJ et al Hip arthroscopy utilization and associated complications: a population-based analysis. J Hip Preserv Surg 2017; 4: 240–9. 28948036 10.1093/jhps/hnx021 PMC5604140

[9] Byrd JW , Jones KS. Prospective analysis of hip arthroscopy with 10-year followup. Clin Orthop Relat Res 2010; 468: 741–6. 19381742 10.1007/s11999-009-0841-7 PMC2816779

[10] Philippon MJ , Briggs KK, Yen YM et al Outcomes following hip arthroscopy for femoroacetabular impingement with associated chondrolabral dysfunction: minimum two-year follow-up. J Bone Joint Surg Br 2009; 91-B: 16–23. 10.1302/0301-620X.91B1.21329 19091999

[11] Egerton T , Hinman RS, Takla A et al Intraoperative cartilage degeneration predicts outcome 12 months after hip arthroscopy. Clin Orthop Relat Res 2013; 471: 593–9. 22992870 10.1007/s11999-012-2594-y PMC3549183

[12] Byrd JWT , Jones KS, Bardowski EA. Influence of Tönnis grade on outcomes of arthroscopy for FAI in athletes: a comparative analysis. J Hip Preserv Surg 2018; 5: 162–5. 29876133 10.1093/jhps/hny011 PMC5961223

[13] Davies O , Grammatopoulos G, Pollard TCB et al Factors increasing risk of failure following hip arthroscopy: a case control study. J Hip Preserv Surg 2018; 5: 240–6. 30393551 10.1093/jhps/hny020 PMC6206686

[14] Outerbridge RE. The etiology of chondromalacia patellae. J Bone Joint Surg Br 1961; 43-B: 752–7. 14038135 10.1302/0301-620X.43B4.752

[15] Philippon M , Schenker M, Briggs K et al Femoroacetabular impingement in 45 professional athletes: associated pathologies and return to sport following arthroscopic decompression. Knee Surg Sports Traumatol Arthrosc 2007; 15: 908–14. 17479250 10.1007/s00167-007-0332-x PMC1950586

[16] Johnston TL , Schenker ML, Briggs KK et al Relationship between offset angle alpha and hip chondral injury in femoroacetabular impingement. Arthroscopy 2008; 24: 669–75. 18514110 10.1016/j.arthro.2008.01.010

[17] Amenabar T , O'Donnell J. Return to sport in Australian football league footballers after hip arthroscopy and midterm outcome. Arthroscopy 2013; 29: 1188–94. 23809453 10.1016/j.arthro.2013.05.001

[18] Beck M , Leunig M, Parvizi J et al Anterior femoroacetabular impingement: part II. Midterm results of surgical treatment. Clin Orthop Relat Res 2004; 418: 67–73. 15043095

[19] Konan S , Rayan F, Meermans G et al Validation of the classification system for acetabular chondral lesions identified at arthroscopy in patients with femoroacetabular impingement. J Bone Joint Surg Br 2011; 93-B: 332–6. 10.1302/0301-620X.93B3.25322 21357954

[20] Kelly BP. Arthroscopic hip anatomy. In: Callaghan JRA, Rubash H (eds). The Adult Hip. Philadelphia: Lippincott Williams & Wilkins, 2004, 78–9.

[21] McCarthy JC , Noble PC, Schuck MR et al The Otto E. Aufranc Award: the role of labral lesions to development of early degenerative hip disease. Clin Orthop Relat Res 2001; 393: 25–37. 10.1097/00003086-200112000-00004 11764355

[22] Lage LA , Patel JV, Villar RN. The acetabular labral tear: an arthroscopic classification. Arthroscopy 1996; 12: 269–72. 8783819 10.1016/s0749-8063(96)90057-2

[23] Seldes RM , Tan V, Hunt J et al Anatomy, histologic features, and vascularity of the adult acetabular labrum. Clin Orthop Relat Res 2001; 382: 232–40. 10.1097/00003086-200101000-00031 11153993

[24] Nepple JJ , Larson CM, Smith MV et al The reliability of arthroscopic classification of acetabular rim labrochondral disease. Am J Sports Med 2012; 40: 2224–9. 22926746 10.1177/0363546512457157

[25] Amenabar T , Piriz J, Mella C et al Reliability of 3 different arthroscopic classifications for chondral damage of the acetabulum. Arthroscopy 2015; 31: 1492–6. 25887376 10.1016/j.arthro.2015.02.029

[26] Walter SD , Eliasziw M, Donner A. Sample size and optimal designs for reliability studies. Stat Med 1998; 17: 101–10. 9463853 10.1002/(sici)1097-0258(19980115)17:1<101::aid-sim727>3.0.co;2-e

[27] Robertson WJ , Kelly BT. The safe zone for hip arthroscopy: a cadaveric assessment of central, peripheral, and lateral compartment portal placement. Arthroscopy 2008; 24: 1019–26. 18760209 10.1016/j.arthro.2008.05.008

[28] James SL , Ali K, Malara F et al MRI findings of femoroacetabular impingement. AJR Am J Roentgenol 2006; 187: 1412–9. 17114529 10.2214/AJR.05.1415

[29] Gwet KL. Handbook of Inter-Rater Reliability. The Definitvie Guide to Measuring the Extent of Agreement among Raters, 2nd edn. Gaithersburg, MD: Advanced Analytics, 2010.

[30] JL F. Measuring nominal scale agreement among many raters. Psychol Bull 1971; 76: 378–82.

[31] Landis JR , Koch GG. The measurement of observer agreement for categorical data. Biometrics 1977; 33: 159–74. 843571

[32] Hariri S , Sochacki KR, Harris AS et al There is poor accuracy in documenting the location of labral and chondral lesions observed during hip arthroscopy. J Exp Orthop 2020; 7: 4. 32008125 10.1186/s40634-020-0221-5 PMC6995460

[33] Cicchetti DV , Feinstein AR. High agreement but low kappa: II. Resolving the paradoxes. J Clin Epidemiol 1990; 43: 551–8. 2189948 10.1016/0895-4356(90)90159-m

