# Supplementary material for: Reliability of the classification of cartilage and labral injuries during hip arthroscopy
Source: J Hip Preserv Surg. 2021 Mar 6;7(3):448–57. doi: 10.1093/jhps/hnaa064 (PMC8081415; doi:10.1093/jhps/hnaa064)

**Appendix 1**

**Observer: ______________**

**Subject ________________**

**Acetabular and Femoral Head Cartilage Injury**


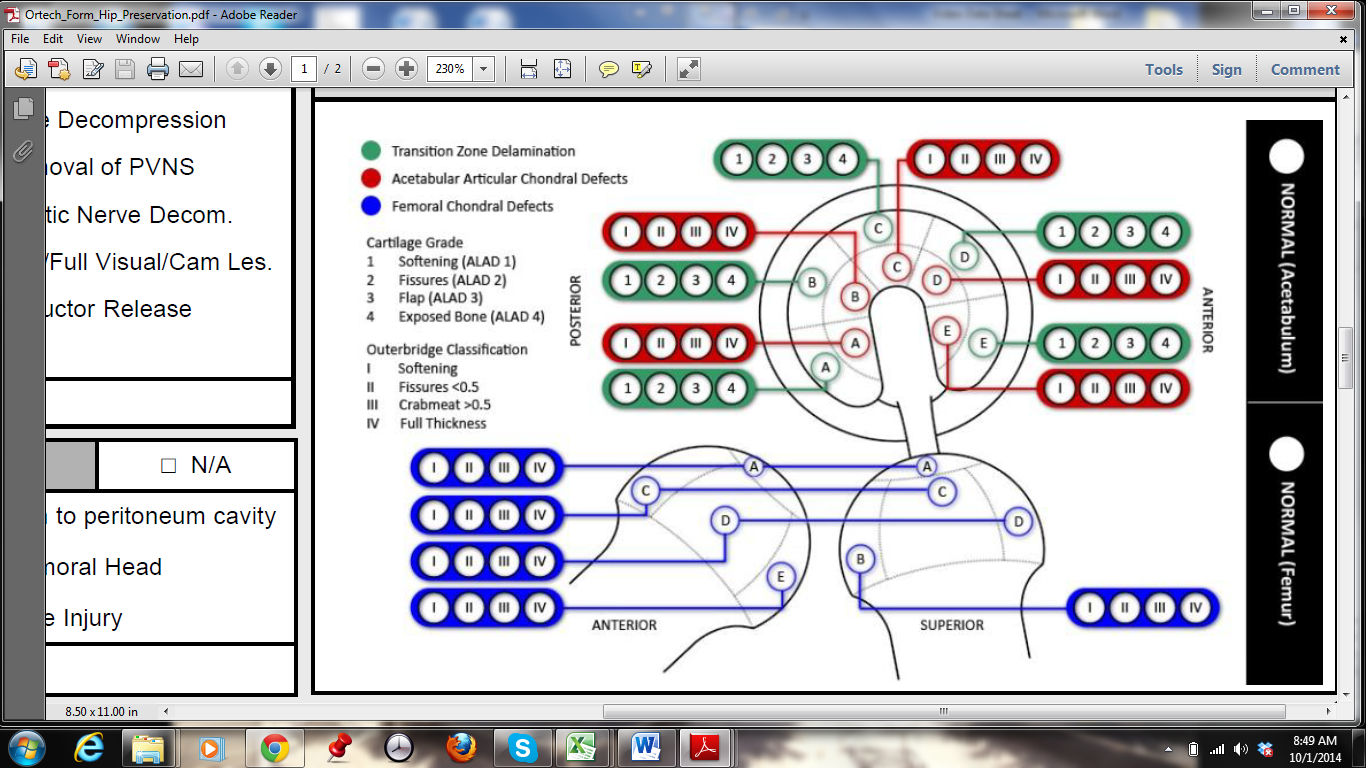


C

For femoral head defects split up the femoral head into quadrants

-10:30 to 1:30 is superior and corresponds to “C”

-1:30 to 4:30 is anterior, and corresponds to “D”

-4:30 to 7:30 is inferior and corresponds to “E”

- 7:30 to 10:30 is posterior and corresponds to “B”

- parafoveal defects correspond to “A”, not clockface


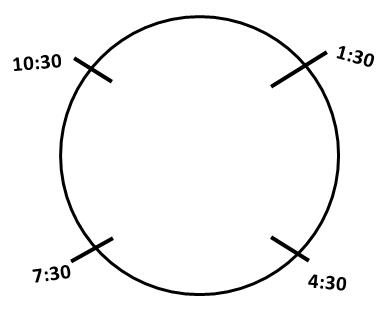


B

D

E

(0) (1) (2) (3) (4)

(0) (1) (2) (3) (4)

**Beck Classification of Cartilage Lesions of Transition Zone**


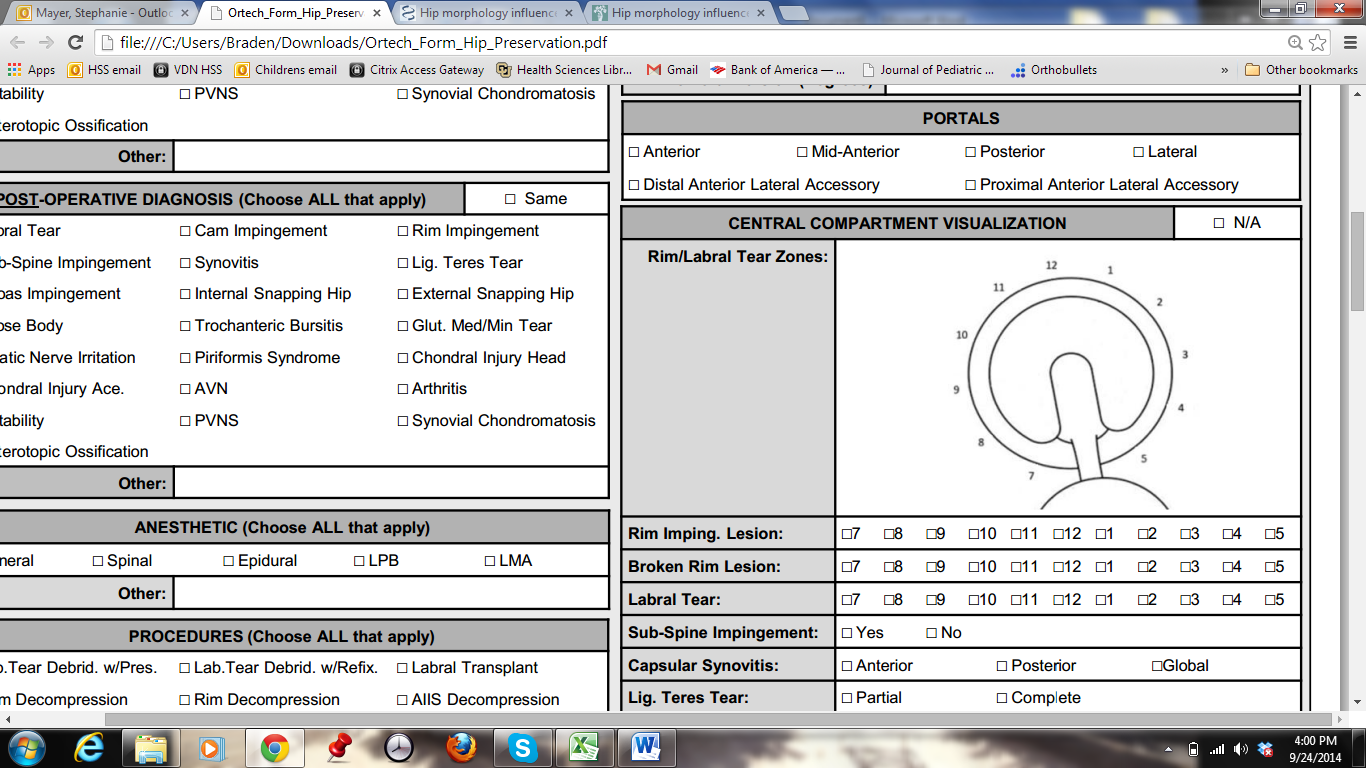


(0) (1) (2) (3) (4)

(0) (1) (2) (3) (4)

(0) (1) (2) (3) (4)


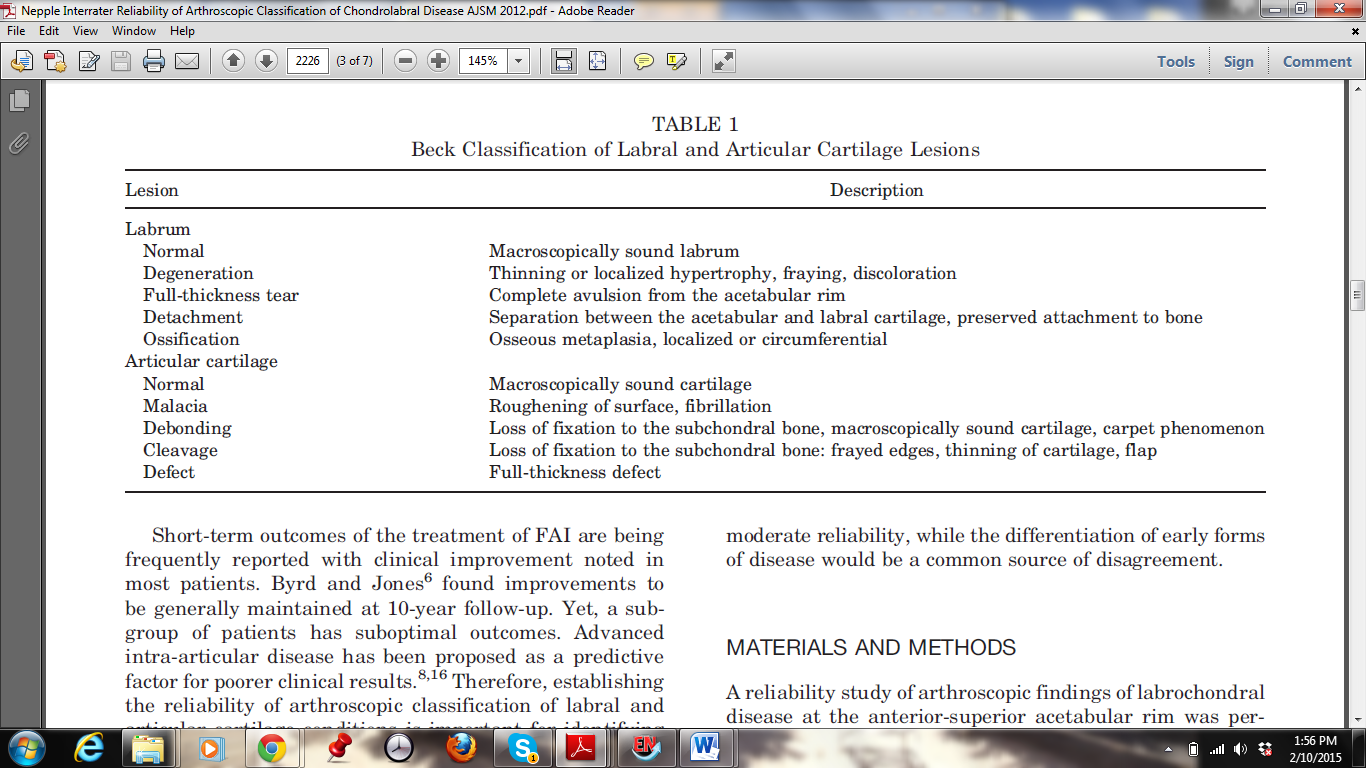


(3)

(4)

(1)

(2)

(0)

**Labral Tear Classification (Beck)**

Normal (0) Macroscopically Normal

Degeneration (1) Thinning or localized hypertrophy, fraying, discoloration

Full Thickness Tear (2) Complete separation from rim

Detachment (3) Separation between acetabular and labral cartilage, preserved attachment to bone

Ossification (4) Osseous metaplasia, localized or circumferential

**Labral Tear Zone on Rim:**


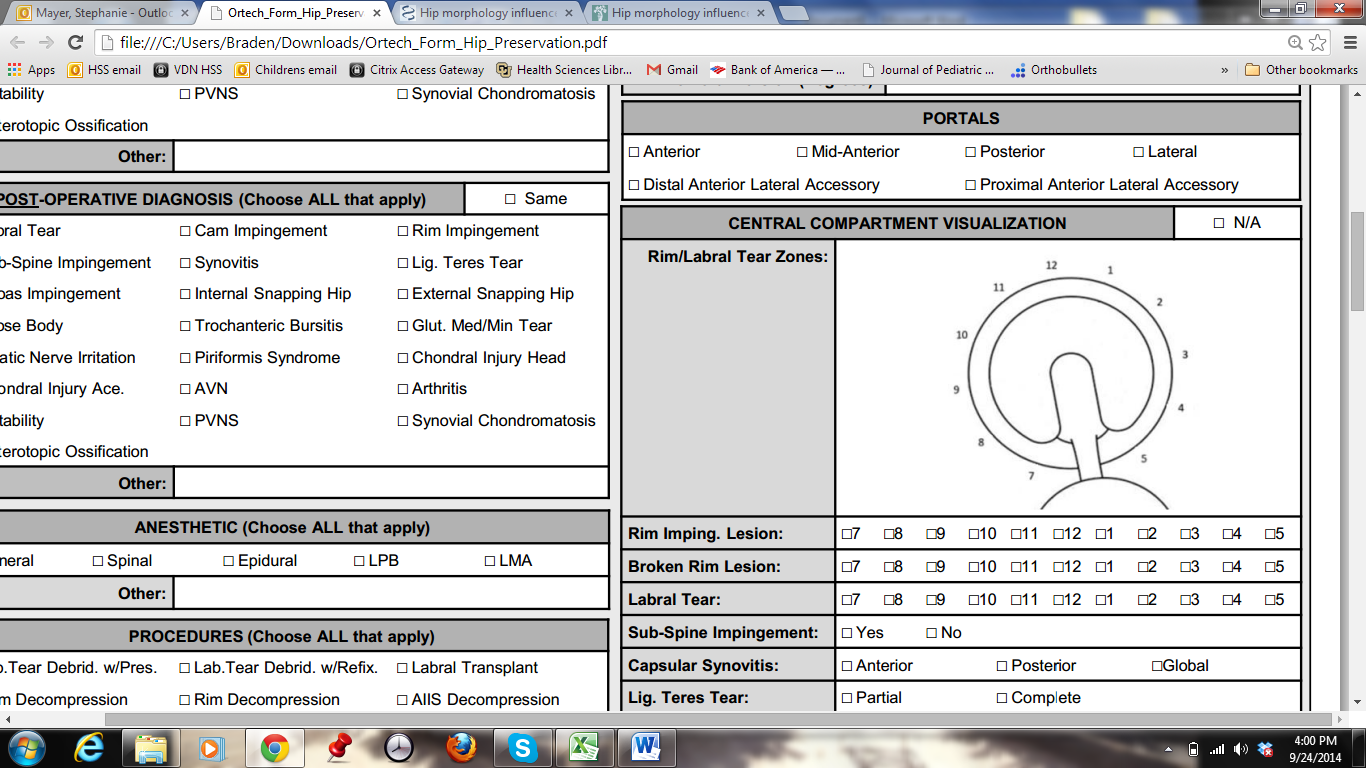

Supplement: hnaa064_Supplementary_Data [file hnaa064_supplementary_data.docx]
